# A Low Dimensional Description of Globally Coupled Heterogeneous Neural Networks of Excitatory and Inhibitory Neurons

**DOI:** 10.1371/journal.pcbi.1000219

**Published:** 2008-11-14

**Authors:** Roxana A. Stefanescu, Viktor K. Jirsa

**Affiliations:** 1 Department of Physics, Florida Atlantic University, Boca Raton, Florida, United States of America; 2 Theoretical Neuroscience Group, Movement Science Institute, CNRS, UMR6233, Marseille, France; 3 Center for Complex Systems and Brain Sciences, Florida Atlantic University, Boca Raton, Florida, United States of America; University College London, United Kingdom

## Abstract

Neural networks consisting of globally coupled excitatory and inhibitory nonidentical neurons may exhibit a complex dynamic behavior including synchronization, multiclustered solutions in phase space, and oscillator death. We investigate the conditions under which these behaviors occur in a multidimensional parametric space defined by the connectivity strengths and dispersion of the neuronal membrane excitability. Using mode decomposition techniques, we further derive analytically a low dimensional description of the neural population dynamics and show that the various dynamic behaviors of the entire network can be well reproduced by this reduced system. Examples of networks of FitzHugh-Nagumo and Hindmarsh-Rose neurons are discussed in detail.

## Author Summary

Nowadays we know that most cognitive functions are not represented in the brain by the activation of a single area but rather by a complex and rich behavior of brain networks distributed over various cortical and subcortical areas. The communication between brain areas is not instantaneous but also undergoes significant signal transmission delays of up to 100 ms, which increase the computation time for brain network models enormously. In order to allow the efficient investigation of brain network models and their associated cognitive capabilities, we report here a novel, computationally parsimonious, mathematical representation of clusters of neurons. Such reduced clusters are called “neural masses” and serve as nodes in the brain networks. Traditional neural mass descriptions so far allowed only for a very limited repertoire of behaviors, which ultimately rendered their description biologically unrealistic. The neural mass model presented here overcomes this limitation and captures a wide range of dynamic behaviors, but in a computationally efficient reduced form. The integration of novel neural mass models into brain networks represents a step closer toward a computational and biologically realistic realization of brain function.

## Introduction

Information processing associated with higher brain functions is believed to be carried out by large scale neural networks [1]–[4]. Significant theoretical and computational efforts have been devoted over the years to understand the dynamical behavior of such networks. While any modeling attempt aspires to preserve the most relevant physical and dynamical characteristics of these networks, certain simplifying hypothesis are usually employed in order to decrease the overwhelming complexity of the problem. In particular, computational models of large scale networks make use of the implicit assumption of “neurocomputational unit”. Such a unit designates a population of thousands of neurons which exhibit a similar behavior. A large scale network is then defined by these units and their interconnections. In order to describe the dynamics of the unit, further assumptions are employed. For instance, the neurons may be regarded as identical entities, the nature and strength of their connections may be neglected and the temporal details of their spiking activity considered irrelevant for the dynamics of the large network. Consequently, a small neural network with these properties will show a very well synchronized dynamics which can be easily captured by a conventional neural mass model (for a comprehensive review see [5] and [6]).

A remarkable amount of scientific work has been devoted to the understanding of the behavior of neural networks when some of these assumptions are dismissed. Many of these studies consider either the inhomogeneities in the network connectivity, or heterogeneous inputs and give a special attention to the synchronized state of the network. Among the first attempts, one may consider the studies on coupled oscillators by Kuramoto [7] who introduced an order parameter capturing the degree of synchronization as a function of the coupling strength or frequency distribution (see [8] and [9] for a comprehensive review). More generally, Pecora et al. [10] (see also Belykh et al. [11]) have derived the master stability equation, serving as a stability condition for the synchronized state of an arbitrary network. Recently, Hennig et al. [12] derive similar conditions considering the connectivity as well as heterogeneous inputs. Another direction for describing the dynamical behavior of such networks involves the derivation of the equations for the synchronized state (described by the mean field or by a synchronization manifold) along with the equations describing the deviations from synchrony [13],[14]. These approaches are suitable only when the deviation from the synchronized state is not very strong. On the other hand, there exists another class of approaches based on mean field theory ([15]; see also [6] for a review). The traditional mean field approaches are incapable of addressing synchronized neural activity, since their basic assumption is that the incoming spike-train to a given neuron in the network is Poissonian and hence uncorrelated. Other dynamical behaviors far from synchrony, such as multi-clustering in the phase for instance, also require expansions of the current approaches. First attempts to do so include the consideration of higher orders in the mean field expansion [16] or mode decompositions of the network dynamics in the phase space [17]. The latter approach by Assisi et al. [17] successfully identified network modes of characteristic behavior, but has been limited to biologically unrealistic situations such as purely excitatory or inhibitory networks and simplistic neuron models. While it is true that strong reductionist assumptions are common (sacrificing dramatically on the biological realism of a network node's dynamics) in large-scale network modeling [18]–[25], these assumptions on the network node's dynamics are usually made adhoc and limit the network dynamics to a small range.

Evidently a reduced small scale network model is desirable to serve as a node in a large scale network simulation whereby displaying a sufficiently rich dynamic repertoire. Here it is of less importance to find a quantitatively precise reduced description of a neural population; rather more importantly, we seek a computationally inexpensive population model (this means typically low-dimensional) which is able to display the major qualitative dynamic behaviors (synchronization, rest state, multi-clustering, etc.) for realistic parameter ranges as observed in the total population of neurons. Here it is also desirable to include biologically more realistic neuron dynamics such as bursting behavior, since novel phenomena on the small scale network level may occur, which need to be captured by the reduced population model.

In this paper we extend the approach by Assisi et al. [17] towards biologically more realistic network architectures including mixed excitatory and inhibitory networks, as well as more realistic neuron models capable of displaying spiking and bursting behavior. Our reduced neural population models not only account for a correct reproduction of the mean field amplitude of the original networks but also capture the most important temporal features of its dynamics. In this way, complex dynamical phenomena such as multi-clustered oscillations, multi-time scale synchronization and oscillation death become available for simulations of large scale neural networks at a low computational cost. We start by investigating first, the main features of the dynamic behavior of a globally coupled heterogeneous neural population comprising both excitatory and inhibitory connections. Then, using mode decomposition techniques, we derive analytically a low dimensional representation of the network dynamics and we show that the main features of the neural population's collective behavior can be captured well by the dynamics of a few modes. Two different neuronal models, a network of FitzHugh-Nagumo neurons and a network of Hindmarsh-Rose neurons are discussed in detail.

## Results

### The Dynamic Behavior of the FitzHugh-Nagumo Neural Population

**Figure 1:**
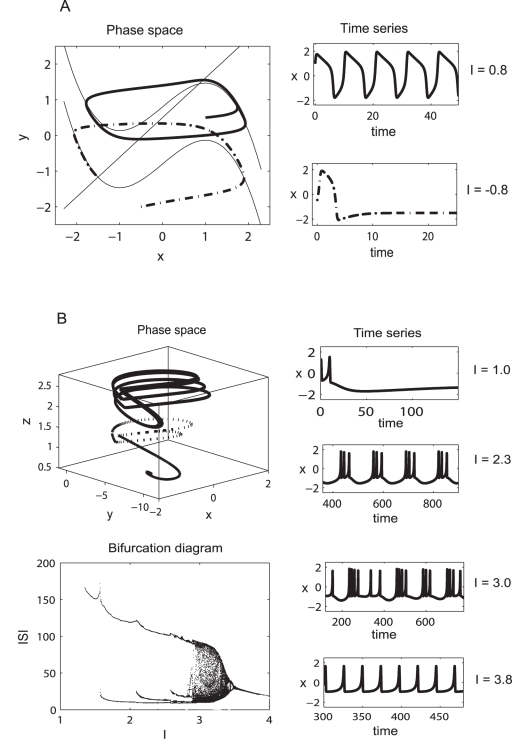
FitzHugh-Nagumo and Hindmarsh-Rose neural models. (A) The intrinsic dynamics of a neuron according to FitzHugh-Nagumo model. Two trajectories in the phase space (left) and their corresponding time series (right) are represented. (B) Hindmarsh-Rose model: the constant parameters used are: (*a* = 1; *b* = 3; *c* = 1; *d* = 5; *s* = 4; *r* = 0.006; *x*_0_ = −1.6). Different dynamic behavior is obtained for different values of the parameter I.

We begin our investigations by considering a mixed population of globally coupled *N*_1_ excitatory and *N*_2_ inhibitory FitzHugh-Nagumo neurons (see Materials and Methods for more details regarding the architecture of the network). The neurons are not identical and differ in the degree of membrane excitability *I_i_*. In normal physiological conditions, this variability may reflect different levels of expression of certain types of receptors [26],[27] or differences in regulatory effects induced by internal [28],[29] or external [30],[31] neuromodulatory processes. Some pathological conditions elicited by specific genetic mutations or by drug abuse are also known to be related to significant modifications of the level of neural membrane excitability [32],[33]. In the framework of theoretical and computational neural modelling, this parameter is usually instantiated by an external current, constant in time, which affects directly the dynamics of the variable describing the neural membrane potential. In general, excitatory or inhibitory subpopulations may be characterized by different parameter distributions. For the purpose of this paper the parameter distribution is called *g*(*I*) and is Gaussian, unless specified differently. The standard deviation of the distribution quantifies the degree of dispersion. The values of the coupling strengths, the dispersion and the mean of the membrane excitability for each subpopulation constitute a parametric space in which the dynamics of the entire population may exhibit various significant characteristics. Because of the multidimensional nature of this parametric space, a complete treatment of the system's dynamics is difficult and some simplifications shall be considered to lower the complexity of the analysis (see the section “Materials and Methods: The architecture of the network” for more details). First, we neglect the coupling within the inhibitory subpopulation (*K*_22_≃0), which is motivated by the small number of inhibitory neurons, second, the coupling strength describing the interactions between the neurons within the excitatory subpopulation *K*_11_ is comparable with the coupling strength *K*_21_ between the excitatory neurons and the neurons in the inhibitory subpopulation; third, rather than allowing arbitrary values for the connectivity strengths *K*_11_ and *K*_12_, we pick a reference value *K*_12_ and manipulate the ratio 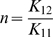. In particular, two parametric regimes may be distinguished: one for which the excitatory coupling is stronger then the inhibitory one (*n*<1), and the opposite situation corresponding to *n*>1. Lastly, we assume that the distributions of the membrane excitability levels in the excitatory and inhibitory subpopulations have the same mean and dispersion parameters. Given the small cortical volume occupied by the neural network considered here, this simplification will be precise, if the concentration changes of neuromodulatory factors influencing the degree of membrane excitability will have identical effects on the two neural subpopulations. Motivated by the simple intrinsic dynamics of the FitzHugh-Nagumo neuron (see Figure 1A), where the stability of the rest state is lost via a Hopf bifurcation, we allow a zero mean for these distributions (*m* = 0) and different degrees of dispersion.

**Figure 2:**
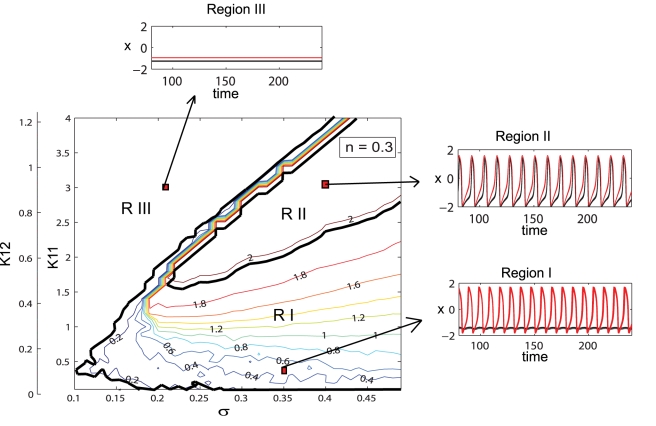
Dynamical regimes of a dominantly excitatory neural population. Contour map of the mean field amplitude calculated for the ratio *n* = 0.3 and mean *m* = 0 are displayed as function of connectivity strengths *K*_11_, *K*_12_ and strength of dispersion *σ*. Three different regions with specific oscillatory behavior are identified. Examples of time series for neurons with low (black) and high (red) value of the *I* parameter are given.

**Figure 3:**
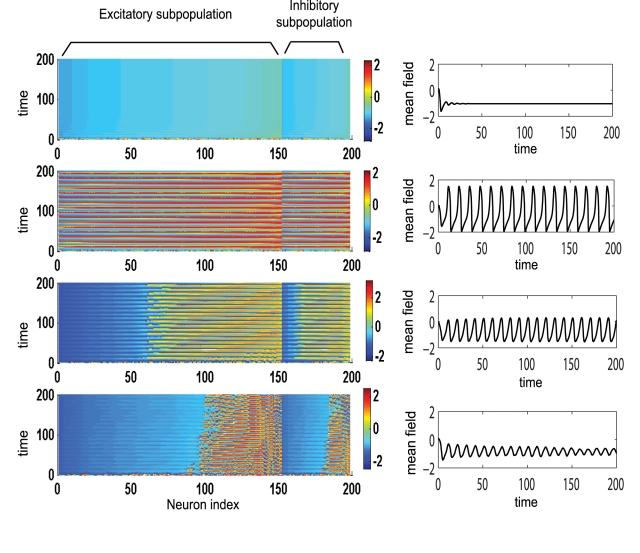
Temporal dynamics of a dominantly excitatory neural population. Left: amplitude color coded time series for all neurons calculated for the following parameter values (starting from bottom to top): *K*_11_ = 0.5 (Region I); *K*_11_ = 0.9 (Region I); *K*_11_ = 2.1 (Region II); *K*_11_ = 3.5 (Region III); for all subfigures *n* = 0.3, *m* = 0 and *σ* = 0.3. Right: the time series of the mean field of the entire population calculated for the same parameters.

Considering these approximations we proceed by investigating how the collective behavior of a population of 200 neurons (150 excitatory and 50 inhibitory neurons) depends on the system's parameters. For small values of the inhibition/excitation ratio (*n*≤0.5) the entire population behaves similar to a purely excitatory population as studied in [17] and summarized in the following. In the parametric space of connectivity strengths *K*_11_, *K*_12_ and dispersion *σ*, three distinct regions can be identified in which the amplitude of the mean field as well as the oscillatory status of the population differs significantly (see Figure 2). The regions are characterized by more than 90% of all neurons showing a behavior particular for a region. In the first region (low values for connectivity strength) the population groups in two clusters, one that will perform large oscillations on the limit cycle and a “quiescent” cluster that performs small oscillations around the fixed point. By increasing the connectivity strength, more and more neurons from the quiescent group will be recruited by the oscillatory cluster, while the oscillations of these neurons become more synchronized. Consequently, the amplitude of the mean field of the population increases. The maximum value is reached in the second region in which all neurons oscillate synchronously. Finally, a third region can be identified for relatively large values of the connectivity strength and small to medium values of dispersion. Here, all the neurons will rest at the stable fixed point hence the mean field amplitude is zero. In Figure 3 we present the amplitude color coded time series for all the neurons calculated for specific parameter values that fall in each of these regions. The neurons in each subpopulation are ordered according to the value of their membrane excitability *I*. Adjacently, we show the time series of the entire population mean field (*X*(*t*)) defined by the equation (6) in Materials and Methods section. Transitions from one region to another can be realized by appropriate changes in the values of parameters. For instance, for large values of the coupling strength a decrease in the value of dispersion parameter may induce the sudden transition from region II (where all neurons are oscillating synchronously) to region III (where all neurons are quiescent) which is sometimes called oscillation death of the neural population [34],[35].

**Figure 4:**
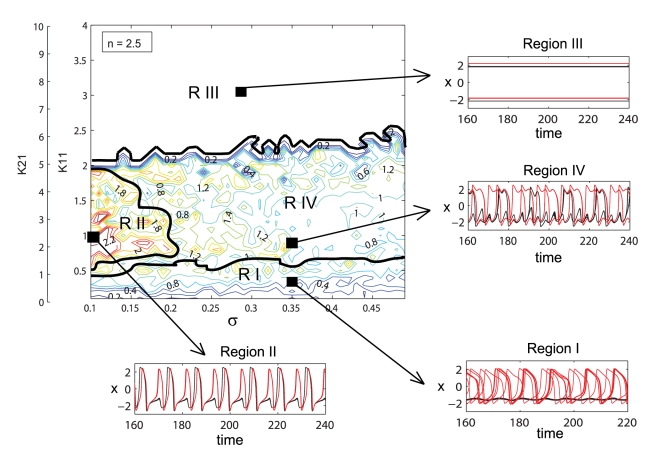
Dynamical regimes of a dominantly inhibitory neural population. Contour map of the mean field amplitude calculated for ratio *n* = 2.5 and mean *m* = 0 are displayed as function of connectivity strengths *K*_11_, *K*_12_ and strength of dispersion *σ*. Four different regions with specific oscillatory behavior are identified. Examples of time series for neurons with low (black) and high (red) value of the *I* parameter are given.

When the ratio of coupling strengths favors a more inhibitory regime, that is for increasing *n*, we observe a significant change in the mean field amplitude landscape (see Figure 4). Once again several regions characterized by different values of the mean field amplitude and qualitatively different oscillatory behavior may be identified.

**Figure 5:**
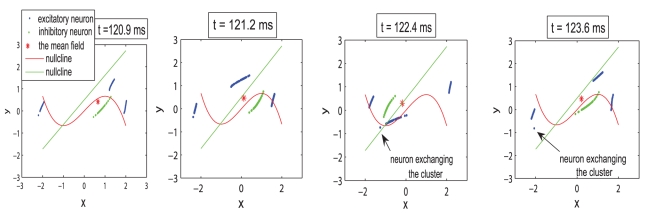
Example of multi-cluster dynamics. Multi-cluster dynamics in phase space with neural cluster exchange obtained for the following parameters: *n* = 1.3; *K*_11_ = 3; *m* = 0; *σ* = 0.3.

**Figure 6:**
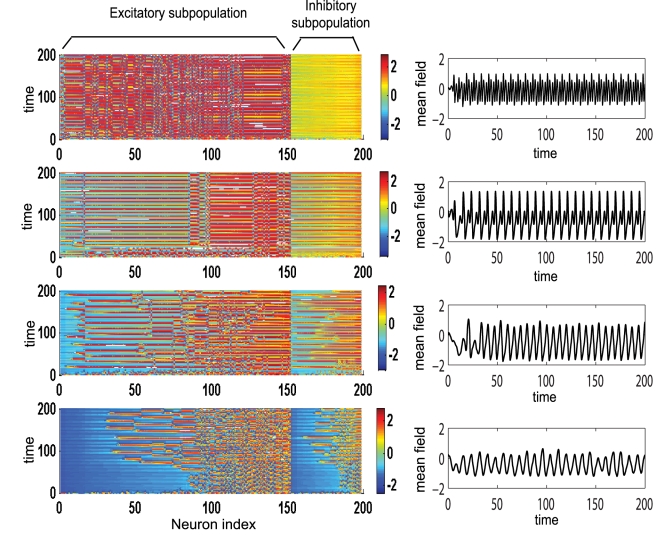
Temporal dynamics of a dominantly inhibitory neural population. Left: Amplitude color coded time series for all neurons calculated for the following parameter values (starting from bottom to top): *K*_11_ = 0.5 (Region I); *K*_11_ = 0.9 (Region I); *K*_11_ = 2.1 (Region II); *K*_11_ = 3.0 (Region III); for all subfigures *n* = 1.3, *m* = 0 and *σ* = 0.3. Right: the time series of the mean field of the entire population calculated for the same parameters.

Regions I and III, corresponding to dynamics of bi-clustering and oscillation death, have been identified and discussed in the previous case as well. In addition we can indicate an interesting region IV that corresponds to values of the mean field amplitude between 0.8 and 1.2. In spite of these lower values, for this regime, all neurons are actually oscillating on limit cycles but clustered in several groups performing, most of the time, antiphase oscillations (Figure 5). While most of the neurons within clusters are synchronized for the entire time, we find also neurons which are exchanging the clusters at various moments. In other words, while the cluster dynamics persists and is invariant, single neurons perform cluster hopping by residing within a given cluster for a longer time duration (≫ then the oscillation period of a cluster) followed by a quick change from one cluster to the other. This complex multi-clustered network dynamics and cluster hopping can not be observed in a purely excitatory population [17]. A final observation regards region II which corresponds to a maximum value of the mean field amplitude. This region is much smaller than the one observed in a mainly excitatory population and is obtained for different values of coupling strengths and dispersion parameters. More than this, the neurons oscillate synchronously only for certain periods of time, while for other periods the dynamics develops in a two cluster regime similar with the one found in region I. The increased complexity of the network dynamics can be observed even for lower values of inhibiton/excitation ratio. In Figure 6, we show amplitude color coded time series for all neurons calculated for a ratio *n* = 1.3, a dispersion *σ* = 0.3 and values for coupling strengths that correspond to different regimes of behavior.

**Figure 7:**
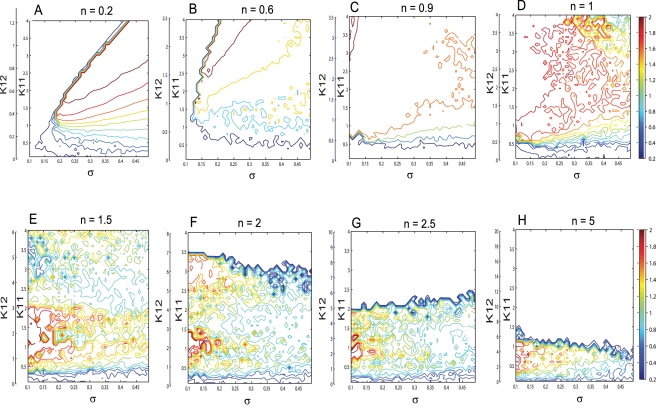
Effects of connectivity on the mean field amplitude. Contour maps of the mean field amplitude of 150 excitatory and 50 inhibitory neurons calculated for different values of the inhibition/excitation ratio ((A) *n* = 0.2, (B) *n* = 0.6, (C) *n* = 0.9, (D) *n* = 1, (E) *n* = 1.5, (F) *n* = 2, (G) *n* = 2.5, (H) *n* = 5). Every map is displayed as a function of the coupling strengths *K*_11_, *K*_12_ and dispersion of membrane excitability distribution *σ*. The mean of the membrane excitability distribution used is *m* = 0.

The abundant dynamic behavior obtained for a mainly inhibitory population may suggest that inhibition does not only play a role in decreasing the firing rate of a certain group of neurons but it may also enrich the global dynamics of the network. The additional dynamical features may be further exploited to accommodate for more complex neural functions. The diverse dynamical features of the network in the extreme situations of mainly excitatory or mainly inhibitory connectivity motivates a further, more systematic analysis for the intermediate regimes. Contour maps of the mean field amplitude have been calculated for increasing values of the inhibition/excitation ratio n and different values of coupling strengths and dispersion parameters (Figure 7). Several important features can be identified. Starting with a mainly excitatory network and increasing the strength of the inhibitory coupling relative to the excitatory one, we can observe a reduction of the region III in favor of the extension of region II, leading to it's complete disappearance for values of n closer to 1 (see Figure 7A–C). When the inhibition strength becomes grater then the excitation (*n*>1), the landscape of the mean field amplitude contour becomes more irregular due to an enlarged sensitivity to the initial conditions. A lower amplitude region starts to emerge at first only for large values of both dispersion and coupling strength (see Figure 7D). Increasing further the value of the ratio n, this region extends towards all the values of the dispersion parameter and smaller values of the coupling strength (see Figure 7E). As discussed above, the mechanism responsible for a lower value of the mean field amplitude is not the decreasing number of oscillatory neurons but the emergence of a multiple cluster dynamics. For even larger values of the inhibition/excitation ratio (*n*>1.5), a new region of zero amplitude of the mean field appears for large coupling strengths (see Figure 7F–H). This region is extended towards smaller values of the connectivity strength leading eventually (for *n*≃8) to the shut down of the entire population for any arbitrary values of the other parameters.

### The Reduced System

**Figure 8:**
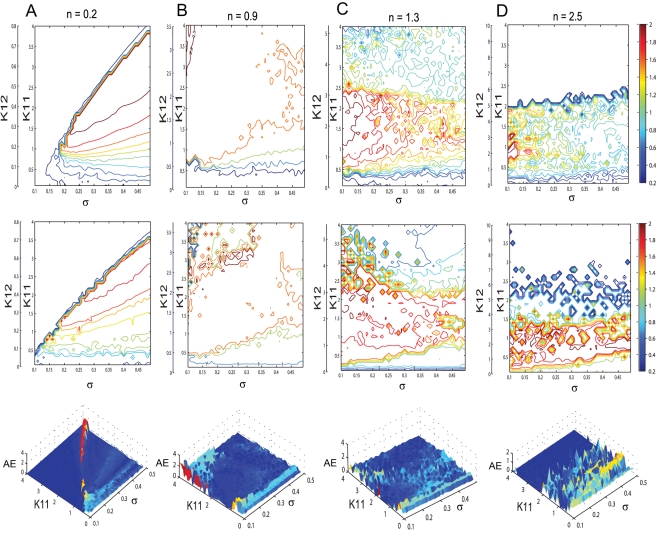
Mean field amplitude landscapes of complete and reduced populations of FitzHugh-Nagumo neurons. Comparison between contour maps of the mean field amplitude obtained using the entire population (upper row) and the reduced system (middle row) for different values of the inhibition/excitation ratio *n* and mean of membrane excitability distribution *m* = 0. Every map is displayed as a function of the connectivity strengths *K*_11_, *K*_12_ and the magnitude of dispersion *σ*. The corresponding surfaces of absolute error (AE) are presented on the bottom row. The values of the normalized mean error (NMAE) calculated for every scenario are: (A) 9.76%, (B) 15.4%, (C) 18.72%, (D) 18.58%.

Proceeding with the mode decomposition technique discussed in detail in Materials and Methods we arrive at a reduced representation of the network dynamics instantiated by the following set of equations:
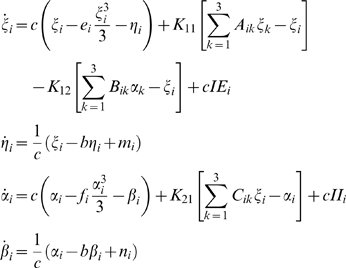
(1)where *i* = 1,2,3 and the quantitative expressions for the coefficients are given in Text S1. Here the variables *ξ_i_*,*η_i_* (and *α_i_*,*β_i_*) describe the dynamics of a given type *i* for the excitatory (and inhibitory) subpopulation of neurons. Using this reduced system we reconstruct in Figure 8 the mean field amplitude for a few parameter scenarios explored in Figure 7. The absolute error of reconstruction (AE), presented in the bottom panels, has been evaluated at every point in the parametric space as the absolute difference between the mean field amplitude (*M*) generated with equations (5) described in Materials and Methods section and the mean field (*Mr*) reconstructed using equations (1).
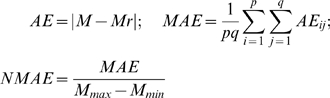
(2)


**Figure 9:**
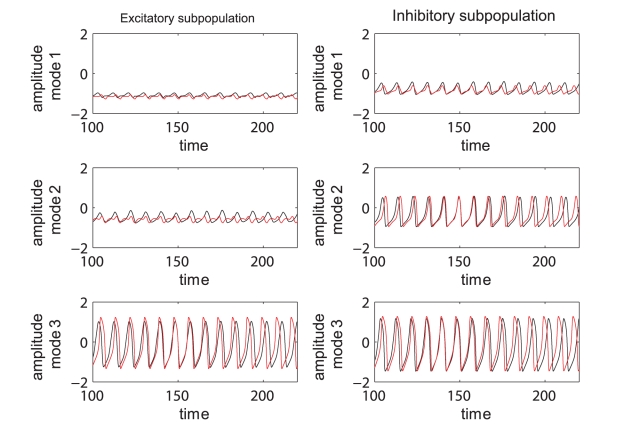
Time series of complete and reduced populations of FitzHugh-Nagumo neurons. Comparison between the temporal series calculated according to the reduced system (black line) and the ones obtained by projecting the time series of the entire system on the modes (red line). The parameters used are the following: *n* = 0.3; *K*_11_ = 0.9; *m* = 0; *σ* = 0.3.

Further, we quantify the overall error for a certain scenario using the normalized mean absolute error (*NMAE*) defined in equation (2), where *p* and *q* stands for the maximum dimensions of the parametric space investigated. Inspecting Figure 8 we note a good reproduction of the main characteristics of the mean field amplitude landscape across all connectivity conditions as judged by visual inspection. The topology of the landscape is captured and all qualitatively different network behaviors are represented. For a more quantitative evaluation, we compute the normalized mean absolute error (*NMAE*) which ranges from 9.76% to a maximum of 18.72%. Most of the significant errors occurs at the borders between regions characterized by different dynamical features (see Regions I to IV identified in Figure 2 and Figure 4). Besides a good approximation of the mean field amplitude of the entire population, one may ask how well is the reduced system capturing the cluster behavior of the full system. In order to address this question we make a comparison between the time series generated by the equations (1) and the ones obtained by direct projection of the time series of the full system on the chosen modes. Examples are given in Figure 9 and in more detail in Figure S1 from the supporting material (Text S1). The modes capture well the amplitude of the corresponding set of neurons, though the phase seems to drift indicative of (potentially nonlinear) frequency contributions compared to the complete network. Simulations consistently show that although the amplitude is correctly reproduced even for a more complicated dynamics (see Figure S1), this might not be always the case with the frequency of the oscillations. In general, one may see periods in which the two time series (stemming from the full and the reduced network simulations) are synchronized followed by periods of less degree in the phase synchronization. This observation may be relevant when transient aspects of phase synchrony play a role in large scale network simulations, but else may not be significant.

### Hindmarsh-Rose Neural Population Model

To reinforce our approach we will consider in the following the case of a mixed population of Hindmarsh-Rose neurons which are known to show spike-burst behavior (see Materials and Methods for more details). As in the previous case analyzed, each neuron is distinguishable from all others due to the value of parameter *I* which may be interpreted as the degree of membrane excitability or an external input.

**Figure 10:**
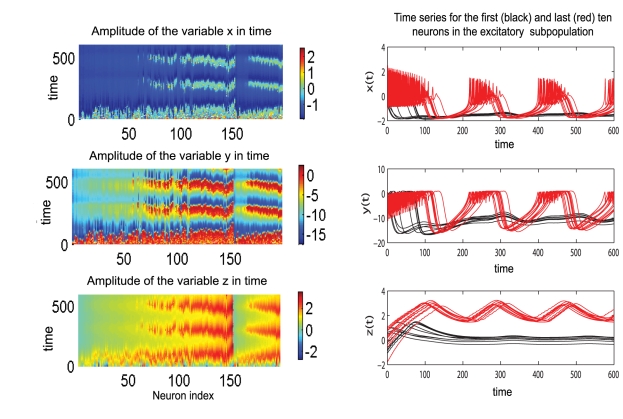
Clustering behavior in a population of Hindmarsh-Rose neurons. Left: Amplitude of the variables *x*,*y*,*z* in time for all neurons ordered according to the value of the parameter *I*. The values of the other parameters are: *n* = 0.5; *K*_11_ = 0.5; *m* = 1.1; *σ* = 0.5; Right: Time series for each variable of the first ten (in black) and the last ten excitatory neurons (in red) calculated for the same parameters as in the left figure.

**Figure 11:**
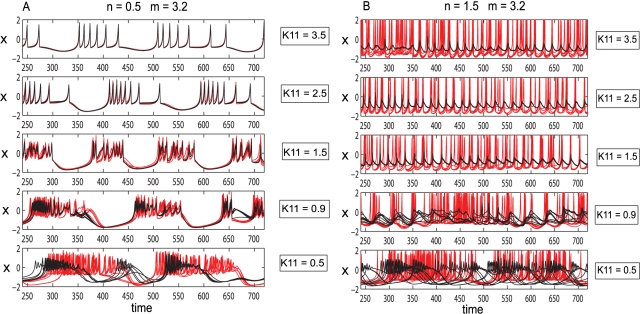
Effects of changing coupling strength in populations of Hindmarsh-Rose neurons. Left: Mainly excitatory coupling: an increase in the coupling strength leads to synchronization within and between the neurons in the excitatory (red) and inhibitory (black) subpopulations. Right: Mainly inhibitory coupling: an increase in the coupling strength induces small amplitude oscillations in the inhibitory subpopulation (black) and a chaotic regime in the excitatory neurons (red).

The bifurcation diagram corresponding to an uncoupled Hindmarsh-Rose neuron (see Figure 1B) suggests that the behavior of the globally coupled mixed population may depend significantly on the mean value (*m*) of the membrane excitability distribution *g*(*I*). Hence, in our attempt to derive a reduced representation of the network dynamics that will capture well the main features of the entire system behavior, we must consider all the possible situations. We start by allowing the mean value for the membrane excitability distribution to be *m* = 1.1. In this case, if uncoupled, part of the neurons will move to the fixed point and part of them will oscillate in a spike-burst manner. The simulations of the globally coupled neural population show indeed a clustering behavior (Figure 10) for low values of the coupling strength (*n* = 0.5; *K*_11_ = 0.5) and large values of the dispersion parameter (*σ* = 0.5). This regime is rapidly left with the increase of coupling strength in favor of a more synchronized dynamics. Unlike the case of the previous model discussed, this two-cluster phenomenon can not be found for other configurations of parameters. An interesting behavior is revealed considering a mean value of *m* = 3.2 and a low value for dispersion *σ* = 0.15. In this condition, if uncoupled, most of the neurons will oscillate chaotically. As a function of the inhibition/excitation ratio, the globally coupled mixed population shows different behaviors. For small values of this ratio (e.g. *n* = 0.5), increase in the excitatory coupling synchronizes the population with a loss of chaotic behavior (see Figure 11A). By contrast, for a large value of this ratio (e.g. *n* = 1.5), an increase in the excitatory coupling will induce small amplitude oscillations in the inhibitory neurons while the excitatory subpopulation exhibits a chaotic regime (see Figure 11B). Simulations show that across all mean values considered, in a mainly excitatory configuration an increase in the connectivity strength will result in a larger degree of burst-spike synchronization within and between subpopulations, and consequently in a larger value of the mean field amplitude. By contrast, in a mainly inhibitory configuration, the increase of coupling strength induces a disorder in the spiking train of oscillation. This dynamics has also been observed in purely excitatory/inhibitory spiking networks with global coupling [13].

### The Reduced System

**Figure 12:**
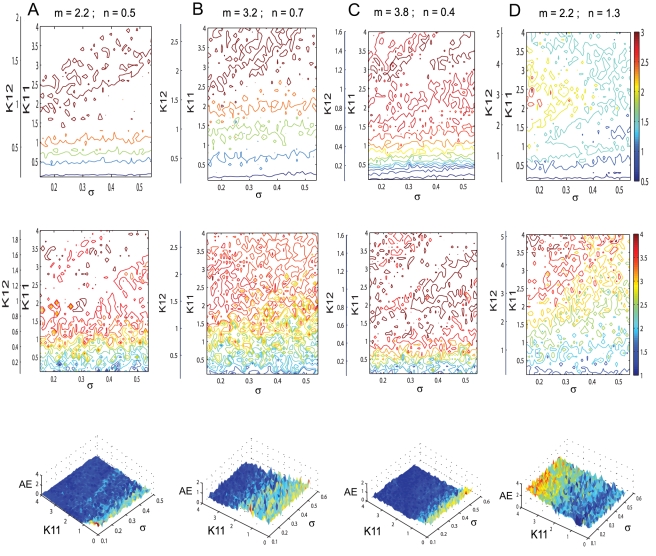
Mean field amplitude landscapes of complete and reduced populations of Hindmarsh-Rose neurons. Comparison between contour maps of the mean field amplitude obtained using the entire population (upper row) and the reduced system (middle row) for different values of the inhibition/excitation ratio *n* and the mean *m* of membrane excitability distribution. Every map is displayed as a function of the connectivity strengths *K*_11_, *K*_12_ and the magnitude of dispersion *σ*. The corresponding surfaces of absolute error (AE) are presented on the bottom row. The values of the normalized mean error (NMAE) calculated for every scenario are: (A) 12.89%, (B) 19.63%, (C) 13.44%, (D) 42.63%.

**Figure 13:**
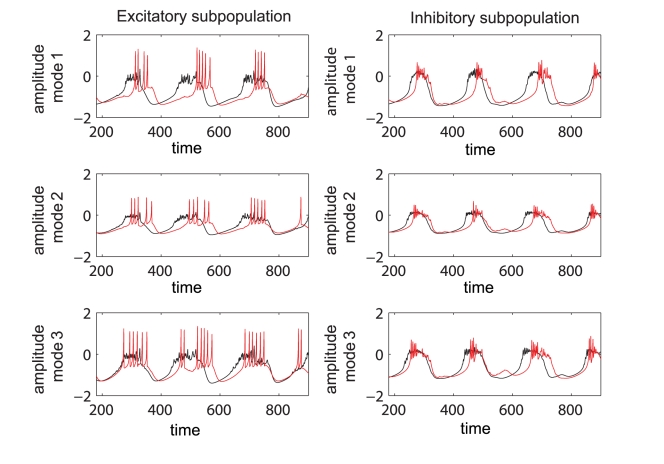
Time series of complete and reduced populations of Hindmarsh-Rose neurons. Comparison between the time series evaluated with the reduced system (red line) and the ones obtained by projecting the time series of the entire system on the chosen modes (black).

As in the previous case, we turn now our attention towards the derivation of a reduced system that can capture the dynamics analyzed above. Applying the mode decomposition technique discussed in Materials and Methods, we find the equations of the reduced representation to be the following:
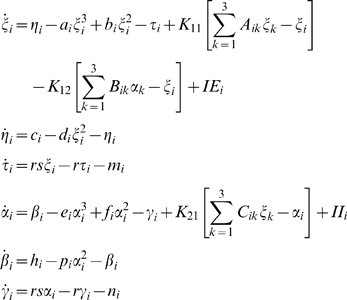
(3)where the analytical expression for the coefficients can be found in Text S1. Here again the index *i* = 1,2,3 codes for the dynamics of type *i*. Using the reduced system we reconstruct in Figure 12 the mean field amplitude contours for different parameter configurations and show the corresponding absolute error (AE) surfaces. As in the case of the previous model analyzed, one may observe a good reproduction of the amplitude landscape for a mainly excitatory population while the mean of the input distribution (*m*) takes various values. The normalized mean absolute error (NMAE) evaluated according to equation (2) takes values between 12.89% and 19.63%. For the case of a mainly inhibitory network the approximation still captures well the amplitude obtained for low coupling strengths but fails for stronger values (*NMAE* = 42.63%). This situation occurs because the excitatory subpopulation oscillates chaotically for this parametric configuration. Projections of the full system's time series (calculated with equations 8) on the modes considered have been compared with the time series of the reduced system (equations 3). The results generated for different parametric scenarios (see Figure 13 as well as in Figure S2 from the Supporting Information (Text S1)) show a very good reproduction of different dynamical features of the system including clustering and spike-burst behavior.

## Discussion

One of the most common assumption employed in computational simulations of large neural networks is the idea that neurons from a small ensemble (sometimes called a “neurocomputational unit”) exhibit a sufficiently similar dynamical behavior. Consequently, the network that instantiates this ensemble, consisting of thousands of excitatory and inhibitory neurons, it is considered to display a synchronized behavior with no other significant temporal features for the dynamics of the large scale network. The main reason for this assumption, is the impractical large computational time arising from too many details considered in the network architecture.

In this paper, we have analyzed the behavior of a neural network that serves as a good example of such a unit, namely a mixed heterogeneous neural population with global coupling, using two neuron models widely employed in theoretical and computational neuroscience. We found that the dynamical features of the network are far more complex then the ones corresponding to synchronized or rest state behavior. The network dynamics depends significantly on the ratio of excitation and inhibition; in fact, the synchronized state can be found only for a mainly excitatory coupling and for a specific range of parameters typically involving a large value for the connectivity strength. On the other hand, a mainly inhibitory neural population may exhibit distinct dynamical features such as multi-clustered behavior (in the case of FitzHugh-Nagumo network) or a chaotic regime (in the case of a Hindmarsh-Rose network). This result suggests that in the real neural networks, inhibition is not only responsible for shutting down the neural activity but may also make alternative dynamic behaviors available to the network, which are unaccessible in a mainly excitatory connectivity. Such dynamical behavior may have a significant contribution to the dynamics of a large scale neural network and consequently, it should be implemented in the computational models. In order to address the problem of the high computational cost of such an implementation, we have further developed a self-consistent low-dimensional neural population model following [17], but incorporating a higher degree of realism. Rather than finding the most appropriate type and number of modes that could minimize a certain error function, we have focussed our attention on constructing a reduced model system which captures the most important network dynamics. First exploratory calculations suggested that a reduction based on the first two modes for the excitatory and inhibitory subpopulation will be insufficient. Significant better results are obtained however, by retaining the first three modes for every subpopulation.

Our detailed analyses demonstrated that the reduced representation manages to recreate correctly the topology of the mean field amplitudes of the original system for various parameter scenarios. To be more specific, more then 80% of the mean field amplitude distributions have been well reproduced across most of the parameter configurations investigated (*NMAE*<20%). In addition, the low dimensional population model is also able to emulate well the main features of the temporal dynamics of the neural network. Certainly the overall performance of the reduced system can be improved quantitatively by considering additional modes in the decomposition. Obviously the choice of modes is an important factor in the development and the efficiency of the representation. Guidance for the particular choice of modes can be taken from cluster analysis in the phase space, in which the minimal number of modes corresponds to the number of clustered neurons in the phase space for a particular parameter configuration. When the modes are chosen to be orthogonal, then the reduced equations decouple linearly. For non-orthogonal modes, the use of a bi-orthogonal mode system will have the same effect. Certain dynamical regimes observed in mixed neural populations, can not be accounted for by a low dimensional system. We have pointed out an example, obtained for the Hindmarsh-Rose neural population in the condition of a mainly inhibitory coupling (see Figure 12D), when for larger coupling strengths, the reduced model fails to reproduce the mean field amplitude of the network. Another example is the situation of cluster hopping of individual neurons. This phenomenon corresponds to a traveling wave in the space spanned by the individual modes, in which a particular neuron shows intermittently the characteristic dynamics of a given mode. To decrease the complexity of analysis of the neural network dynamical behavior in the multidimensional parameter space, we have employed certain assumptions. For instance, we have ignored the connectivity between the neurons within the inhibitory subpopulation and we have assumed similar values for the mean of the membrane excitability distributions for both excitatory and inhibitory subpopulations. Following the method outlined in the paper, we can derive reduced representation for the original system even when these assumptions are removed, in fact ensuring a convenient generality of the procedure.

From a more general perspective, despite its limitations, our approach may offer a viable alternative to the neural mass models currently used in the literature. We emphasize here that because of the “near to synchrony” assumption, neural mass models can not capture complex dynamical features such as multi-clustering, oscillator death or multi-time scale synchronization. By comparison, our model offers the possibility to account for such features at a very low computational cost. Therefore, the reduced representation discussed in this paper qualifies as a good candidate for a “neural unit” in computational simulations of large scale neural networks.

## Materials and Methods

### The Architecture of the Network

**Figure 14:**
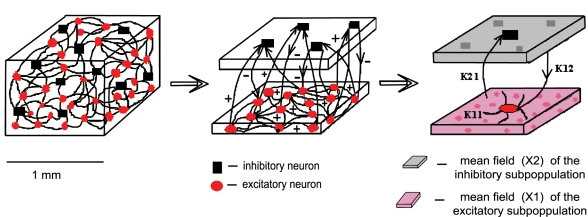
Schematic of the network architecture. In red we represent the excitatory neurons and in black the inhibitory ones. The mean field *X*_1_ of the excitatory subpopulation (pink) is driving every neuron, while the mean field *X*_2_ of the inhibitory subpopulation (gray) affects only the excitatory neurons.

To reflect biophysically realistic architectures, we model the connectivity in the mixed population as follows: every neuron from the excitatory subpopulation is linearly coupled with any other neuron; each inhibitory neuron is driven only by the coupling with its excitatory partners [36] (for a schematic cartoon see Figure 14). This architecture is motivated by the presence of roughly 90% excitatory and 10% inhibitory neurons in a typical volume element of cortex [37]. The linear coupling captures precisely electric coupling through gap junctions and approximatively synaptic coupling when the average population activity is constrained to a small signal range. We wish to emphasize that the connectivity is instantaneous, hence our network cannot account for any phenomena related to synaptic transmission delay. The latter become relevant when considering large scale networks. For small networks as considered here, the transmission delays are negligible. In the brain, the communication between any two neurons in the cortex is achieved typically via monosynaptic couplings. The position on the dendritic tree, the dimension of the synaptic terminal and the distribution and type of synaptic receptors are just a few factors that can determine the efficacy of every synapse. Here we consider averages of all these properties over each neural subpopulation and we absorb them in our models by the connectivity strength parameters *K_ij_* with *i*, *j* = 1,2. Regarding anatomical constraints, we make the following considerations: The strength of connectivity between neurons within the excitatory subpopulation (*K*_11_) may differ from the connectivity strength between excitatory and inhibitory neurons (*K*_12_). We capture their interdependence by the ratio 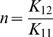. The excitatory-inhibitory couplings may not be necessarily bidirectional hence another value of the connectivity between inhibitory and excitatory neurons (*K*_21_) is considered separately. Finally, we neglect any possible couplings within the inhibitory subpopulation (*K*_22_≃0), reflecting the small probability of interneuron-interneuron connections due to the characteristic sparseness of these neurons in a small cortex volume (see [38] for a comprehensive review).

### Neural Network of FitzHugh-Nagumo Neurons

FitzHugh-Nagumo model [39],[40] provides one of the simplest and most widely used representation of an excitable neural system. The dynamics is governed by two differential equations:
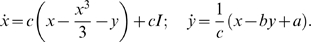
(4)where the variables *x* and *y* evolves on a fast and respectively slow time scale. According to the value of the parameter I which may be considered either an external input or the neural membrane excitability, the system may oscillate (on a limit cycle) or reach an equilibrium state (a stable fixed point) (see Figure 1A). This parameter determines the position of the cubic nullcline and through such, the fixed points and their nature.

Employing this model for the network following the architecture described above, and considering as well the average activity *X_i_* of the *i_th_* subpopulation, we can describe the dynamics of the system by the following set of equations:
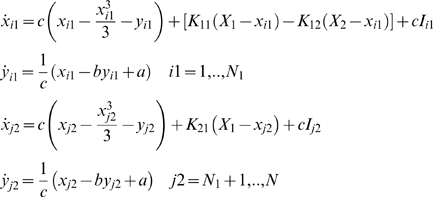
(5)where *N* = *N*_1_+*N*_2_ is the total number of neurons, *a* = 0.45, *b* = 0.9, *c* = 3 are constants and
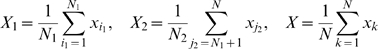
(6)are the mean fields of the excitatory and inhibitory subpopulations, respectively the mean field of the entire neural population.

The first two equations describe the time dependence of the fast and slow variables for every excitatory neuron while the last two equations specify the dynamics of the same variables corresponding to every inhibitory neuron.

### Neural Network of Hindmarsh-Rose Neurons

The Hindmarsh-Rose model [41],[42] is another example of excitable system often employed to account for a more complex phenomenon, namely neuronal bursting oscillations. The model consists of a set of three differential equations:
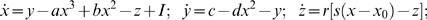
(7)where the variables *x* and *y* are evolving on a fast time scale while *z* is a slow variable. As a function of the parameter *I*, the system may exhibit a fixed point dynamics (*I*<1.32), a spike burst behavior (*I*>1.32) with a chaotic regime for 2.92<*I*<3.40 and a simple oscillatory dynamics for *I*>3.4 (see Figure 1B).

This model has often been considered in studies regarding neural systems showing transitions from rest state to a firing state consisting in a burst of several spikes [13],[43],[44].

Employing the same connectivity model as the one described in the first section, we can describe the dynamics of the population with the following set of equations:
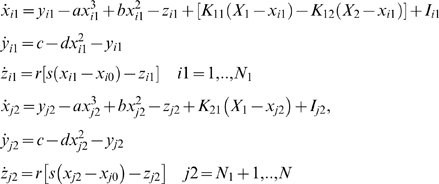
(8)where *N* = *N*_1_+*N*_2_ is the total number of neurons, *a* = 1; *b* = 3; *c* = 1; *d* = 5; *s* = 4; *r* = 0.006; *x*_0_ = −1.6 are constants and
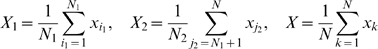
(9)are the mean fields of the excitatory and inhibitory subpopulations, respectively the mean field of the entire neural population.

The first three equations describe the time evolution for every neuron in the excitatory subpopulation while the remaining equations account for the dynamics of the neurons in the inhibitory subpopulation.

### The Reduced System of the Neural Population

We start by recalling that the distinction between the neurons in the same subpopulation is due solely to the value of the *I_i_* parameter. Thus, we can consider an ordering of the neurons according to the magnitude of this parameter such that *I_i_*_+1_>*I_i_*. The state vector for the *i_th_* and *j_th_* neuron in the excitatory respectively inhibitory subpopulation may be reformulated in terms of this parametric dependence as follows:
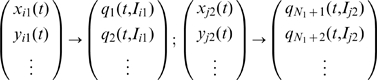
(10)


For a large enough system, the sets {*I_i_*_1_} and {*I_j_*_2_} can be treated as a continuous variable 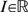 and each subnetwork state vector as a continuous vector field:
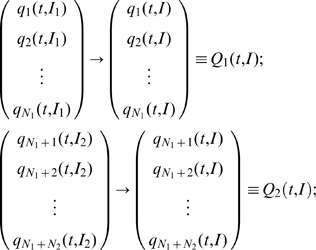
(11)


Considering the fact that for a Gaussian distribution the first moment is equal with its mean, we can reformulate the mean field amplitude for the excitatory and inhibitory subpopulation as follows:
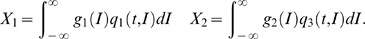
(12)where *g*_1_(*I*) and *g*_2_(*I*) are the excitatory respectively inhibitory parametric distributions.

This reformulation of the network state vector in a continuous parametric space allows as to use mode decomposition techniques to find the dominant patterns of the behavior of the entire population. We begin by expressing the state vector of each subpopulation as a superposition of a finite number of modes. Given the fact that the initial distribution of the *I* parameter for each subpopulation may lie in a different range of values, the significant modes for each subpopulation may differ. Hence, we will consider the set of modes *v_i_* for the excitatory subpopulation and the set *u_j_* for the inhibitory one with their corresponding time dependent coefficients. In this framework, the state vectors can be written as:
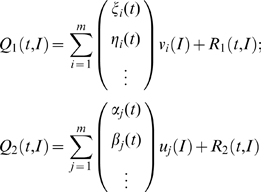
(13)where *R*_1_(*t*,*I*) and *R*_2_(*t*,*I*) represents the residuals of the decomposition accounting for the spatiotemporal dynamics not captured by the first *m* modes.

In general, the modes considered above are not orthogonal. However, an appropriate adjoint basis 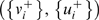 may be constructed to insure the biorthogonality condition.
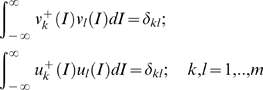
(14)


Commonly, arbitrary mode decomposition techniques may be chosen, which have the property to minimize an error function. However to allow for a functional interpretation of the modes it is desirable, if the modes correspond to characteristic clusters in phase space as shown in Figure 5. Simply put, neurons with a higher firing threshold will be less likely to be found in the strongly firing (i.e. oscillatory) cluster and more likely to be a member of the quiescent cluster. For this reason, the partitioning of the I axis into disjunct, non-overlapping modes is a promising first approach.

In the following we utilize three modes per population type, in which we distinguish regimes of parameter I corresponding to small, medium and high I-values. In this particular case, the modes have been chosen to be approximatively non-overlapping rectangular functions (see Figure S3 from the supporting material (Text S1) for more details).

Introducing equations (12) and (13) into (5) and (8), multiplying with the adjoint of each mode and integrating over the entire space we obtain the equations (1) and (3) (see Results section) that describes the temporal evolution of the mode coefficients corresponding to the FitzHugh-Nagumo neural population, respectively the Hindmarsh-Rose network. We emphasize here, that the cross terms resulting from the nonlinearities in equations (5) and (8) disappear because of the bi-orthogonality condition instantiated by equation (12).

## Supporting Information

Figure S1 Time series of complete and reduced populations of FitzHugh-Nagumo neurons evaluated for different parametric regimes. Comparison between the temporal series calculated according to the reduced system described by equations (1) (red line) and the ones obtained by projecting the time series of the entire system (equations (5)) on the modes (black line). The following parametric regimes are considered: (A) *n* = 0.3; *K*_11_ = 1.2; *σ* = 0.3; (B) *n* = 0.6; *K*_11_ = 2; *σ* = 0.25; (C) *n* = 1.5; *K*_11_ = 1.5; *σ* = 0.3.
(5.00 MB EPS)

Figure S2 Time series of complete and reduced populations of Hindmarsh-Rose neurons evaluated for different parametric regimes. Comparison between the temporal series calculated according to the reduced system described by equations (3) (red line) and the ones obtained by projecting the time series of the entire system (equations (8)) on the modes (black line). The following parametric regimes are considered: (A) *m* = 1.2; *n* = 0.8; *K*_11_ = 0.8; *σ* = 0.35; (B) *m* = 2.2; *n* = 1.3; *K*_11_ = 0.6; *σ* = 0.25; (C) *m* = 3.2; *n* = 0.4; *K*_11_ = 1.5; *σ* = 0.4; (D) *m* = 3.8; *n* = 0.5; *K*_11_ = 2.3; *σ* = 0.3.
(4.68 MB EPS)

Figure S3 Example of modes of decomposition and membrane excitability parametric distribution used for the excitatory subpopulation. (A) Values of the *I* parameter for every neuron versus initial neural index. (B) Ordered values of the *I* parameter for every neuron versus reassigned neural index. The three modes used in decomposition analysis: v1(*I*)(blue), v2(*I*)(green), v3(*I*)(red) are superimposed on the ordered *I* parametric distribution. (C) Histogram of the Gaussian distribution of membrane excitability. (D) The modes used in the decomposition ananlysis are superimposed on the integrable form of the Gaussian parametric distribution.
(1.07 MB EPS)

Text S1 Supporting information
(0.06 MB PDF)
