# Supplementary material for: A Low Dimensional Description of Globally Coupled Heterogeneous Neural Networks of Excitatory and Inhibitory Neurons
Source: PLoS Comput Biol. 2008 Nov 14;4(11):e1000219. doi: 10.1371/journal.pcbi.1000219 (PMC2574034; doi:10.1371/journal.pcbi.1000219)
Supplement: Text S1 — Supporting information (0.06 MB PDF) [file pcbi.1000219.s004.pdf]

Text S1

The coefficients of the reduced model of a mixed population of FitzHugh-Nagumo neurons are the following:

$$A_{ik} = \int_{-\infty}^{\infty} g_1(z')v_k(z')dz' \int_{-\infty}^{\infty} v_i^+(z)dz; \qquad B_{ik} = \int_{-\infty}^{\infty} g_2(z')u_k(z')dz' \int_{-\infty}^{\infty} v_i^+(z)dz;$$

$$C_{ik} = \int_{-\infty}^{\infty} g_1(z')v_k(z')dz' \int_{-\infty}^{\infty} u_i^+(z)dz;$$

$$e_i = \int_{-\infty}^{\infty} v_i^+(z)v_i^3(z)dz; \qquad IE_i = \int_{-\infty}^{\infty} zv_i^+(z)dz; \qquad m_i = a\int_{-\infty}^{\infty} v_i^+(z)dz;$$

$$f_i = \int_{-\infty}^{\infty} u_i^+(z)u_i^3(z)dz; \qquad II_i = \int_{-\infty}^{\infty} zu_i^+(z)dz; \qquad n_i = a\int_{-\infty}^{\infty} u_i^+(z)dz;$$

A numerical example calculated considering Gaussian input distributions of mean $m = 0$ and standard deviation $\sigma = 0.35$ reads:

$$A = \begin{bmatrix} 0.3262 & 0.5398 & 0.3398 \\ 0.2150 & 0.3558 & 0.2240 \\ 0.2841 & 0.4701 & 0.2960 \end{bmatrix}; \qquad B = \begin{bmatrix} 0.3963 & 0.5268 & 0.2977 \\ 0.2612 & 0.3473 & 0.1963 \\ 0.3451 & 0.4589 & 0.2593 \end{bmatrix};$$

$$C = \begin{bmatrix} 0.2681 & 0.4436 & 0.2792 \\ 0.2092 & 0.3461 & 0.2179 \\ 0.3274 & 0.5418 & 0.3411 \end{bmatrix};$$

$$e = \begin{bmatrix} 1.3437 & 3.0923 & 1.7714 \end{bmatrix}; \qquad f = \begin{bmatrix} 1.9898 & 3.2674 & 1.3338 \end{bmatrix};$$

$$IE = \begin{bmatrix} -0.4535 & 0.0046 & 0.3397 \end{bmatrix}; \qquad II = \begin{bmatrix} -0.2664 & 0.0155 & 0.4830 \end{bmatrix};$$

$$m = \begin{bmatrix} 0.3882 & 0.2559 & 0.3381 \end{bmatrix}; \qquad n = \begin{bmatrix} 0.3190 & 0.2489 & 0.3896 \end{bmatrix};$$

The coefficients of the reduced model of a mixed population of Hindmarsh-Rose neurons are the following:

$$A_{ik} = \int_{-\infty}^{\infty} g_1(I')v_k(I')dI' \int_{-\infty}^{\infty} v_1^{+}(I)dI; \qquad B_{ik} = \int_{-\infty}^{\infty} g_2(I')u_k(I')dI' \int_{-\infty}^{\infty} v_i^{+}(I)dI;$$

$$C_{ik} = \int_{-\infty}^{\infty} g_1(I')v_k(I')dI' \int_{-\infty}^{\infty} u_i^{+}(I)dI;$$

$$a_i = \int_{-\infty}^{\infty} av_i^{+}(I)v_i^3(I)dI; \quad b_i = \int_{-\infty}^{\infty} bv_i^{+}(I)v_i^2(I)dI; \quad IE_i = \int_{-\infty}^{\infty} Iv_i^{+}(I)dI;$$

$$c_i = \int_{-\infty}^{\infty} cv_i^{+}(I)dI; \qquad d_i = \int_{-\infty}^{\infty} dv_i^{+}(I)dI; \qquad m_i = \int_{-\infty}^{\infty} rsx_0v_i^{+}(I)dI;$$

$$e_i = \int_{-\infty}^{\infty} au_i^{+}(I)u_i^3(I)dI; \quad f_i = \int_{-\infty}^{\infty} bu_i^{+}(I)u_i^2(I)dI; \quad II_i = \int_{-\infty}^{\infty} Iu_i^{+}(I)dI;$$

$$h_i = \int_{-\infty}^{\infty} cu_i^{+}(I)dI; \qquad p_i = \int_{-\infty}^{\infty} du_i^{+}(I)dI; \qquad n_i = \int_{-\infty}^{\infty} rsx_0u_i^{+}(I)dI;$$

A numerical example calculated considering Gaussian input distributions of mean $m = 2.2$ and standard deviation $\sigma = 0.3$ reads:

$$A = \begin{bmatrix} 0.3126 & 0.5170 & 0.3301 \\ 0.2078 & 0.3437 & 0.2195 \\ 0.3138 & 0.5190 & 0.3313 \end{bmatrix}; \qquad B = \begin{bmatrix} 0.3691 & 0.4706 & 0.3505 \\ 0.2454 & 0.3128 & 0.2330 \\ 0.3705 & 0.4723 & 0.3518 \end{bmatrix};$$

$$C = \begin{bmatrix} 0.2978 & 0.4925 & 0.3144 \\ 0.1869 & 0.3092 & 0.1974 \\ 0.3115 & 0.5151 & 0.3289 \end{bmatrix};$$

$$a = \begin{bmatrix} 1.6545 & 3.7432 & 1.6422 \end{bmatrix}; \qquad e = \begin{bmatrix} 1.8234 & 4.6275 & 1.6670 \end{bmatrix};$$

$$b = \begin{bmatrix} 3.8588 & 5.8042 & 3.8444 \end{bmatrix}; \qquad f = \begin{bmatrix} 4.0510 & 6.4535 & 3.8734 \end{bmatrix};$$

$$c = \begin{bmatrix} 0.7774 & 0.5169 & 0.7804 \end{bmatrix}; \qquad p = \begin{bmatrix} 0.7405 & 0.4649 & 0.7745 \end{bmatrix};$$

$$IE = \begin{bmatrix} 1.3650 & 1.1328 & 2.0520 \end{bmatrix}; \qquad II = \begin{bmatrix} 1.3491 & 1.0241 & 2.0233 \end{bmatrix};$$

$$d = \begin{bmatrix} 6.4313 & 9.6737 & 6.4074 \end{bmatrix}; \qquad p = \begin{bmatrix} 6.7517 & 10.7558 & 6.4557 \end{bmatrix};$$

$$m = \begin{bmatrix} -0.0299 & -0.0198 & -0.0300 \end{bmatrix}; \quad n = \begin{bmatrix} -0.0284 & -0.0179 & -0.0297 \end{bmatrix};$$

where the index i takes the values 1,2 and 3.

Figure Legends

Figure 1: **Time series of complete and reduced populations of FitzHugh-Nagumo neurons evaluated for different parametric regimes.** Comparison between the temporal series calculated according to the reduced system described by equations (1) (red line) and the ones obtained by projecting the time series of the entire system (equations (5) on the modes (black line). The following parametric regimes are considered: Figure S1A - $n = 0.3; K_{11} = 1.2; \sigma = 0.3$; Figure S1B - $n = 0.6; K_{11} = 2; \sigma = 0.25$; Figure S1C - $n = 1.5; K_{11} = 1.5; \sigma = 0.4$; Figure S1D - $n = 2.1; K_{11} = 0.8; \sigma = 0.3$.

Figure 2: **Time series of complete and reduced populations of Hindmarsh-Rose neurons evaluated for different parametric regimes.** Comparison between the temporal series calculated according to the reduced system described by equations (3) (red line) and the ones obtained by projecting the time series of the entire system (equations (8)) on the modes (black line). The following parametric regimes are considered: Figure S2A - $m = 1.2; n = 0.8; K_{11} = 0.8; \sigma = 0.35$; Figure S2B - $m = 2.2; n = 1.3; K_{11} = 0.6; \sigma = 0.25$; Figure S2C - $m = 3.2; n = 0.4; K_{11} = 1.5; \sigma = 0.4$; Figure S2D - $m = 3.8; n = 0.5; K_{11} = 2.3; \sigma = 0.3$.

Figure 3: **Example of modes of decomposition and membrane excitability parametric distribution used for the excitatory subpopulation.** Figure S3A - Values of the I parameter for every neuron versus initial neural index; Figure S3B - Ordered values of the I parameter for every neuron versus reassigned neural index. The three modes used in decomposition analysis: $v_1(I)$ (blue),$v_2(I)$ (green) and $v_3(I)$ (red) are superimposed on the ordered I parametric distribution; Figure S3C - Histogram of the Gaussian distribution of membrane excitability ; Figure S3D - The modes used in the decomposition analysis are superimposed on the integrable form of the Gaussian parametric distribution.
